# A Versatile Reporter Platform for Evaluating HDR- and NHEJ-Based Genome Editing in Airway Epithelial Cell Cultures Using an rAAV Vector

**DOI:** 10.3390/v17060821

**Published:** 2025-06-06

**Authors:** Soo Yeun Park, Zehua Feng, Xiujuan Zhang, Yinghua Tang, Donovan Richart, Kai E. Vorhies, Jianming Qiu, John F. Engelhardt, Ziying Yan

**Affiliations:** 1 Department of Anatomy and Cell Biology, Carver College of Medicine, University of Iowa, Iowa City, IA 52242, USA; 2 Division of Pulmonary, Allergy, Critical Care & Sleep Medicine, School of Medicine, University of Alabama at Birmingham, Birmingham, AL 35294, USA; 3 Department of Microbiology, Molecular Genetics and Immunology, University of Kansas Medical Center, Kansas City, KS 66160, USA

**Keywords:** adeno-associated virus vector, gene editing, reporter cell lines, airway epithelial cultures

## Abstract

Therapeutic gene editing strategies utilize endogenous DNA repair pathways—nonhomologous end joining (NHEJ) or homology-directed repair (HDR)—to introduce targeted genomic modifications. Because HDR is restricted to dividing cells, whereas NHEJ functions in both dividing and non-dividing cells, NHEJ-based approaches are better suited for in vivo gene editing in the largely post-mitotic airway epithelium. Homology-independent targeted insertion (HITI), an NHEJ-based method, offers a promising strategy for cystic fibrosis (CF) gene therapy. Here, we applied HITI to drive the expression of a promoterless reporter through an exon trap strategy in both proliferating airway basal cells and well-differentiated primary airway epithelial cultures derived from transgenic ROSA^mTmG^ ferrets. We also established a versatile human gene editing reporter (GER) airway basal cell line capable of multipotent differentiation, enabling real-time visualization of editing outcomes and the quantitative assessment of HDR- and NHEJ-based editing efficiencies. Together, these platforms provide easily accessible tools for optimizing genome editing strategies in the respiratory epithelium and advancing clinically relevant delivery strategies for CF gene therapy.

## 1. Introduction

Cystic fibrosis (CF) is a monogenic disorder caused by mutations in the *CFTR* gene, which encodes the cystic fibrosis transmembrane conductance regulator (CFTR) protein [1,2]. This life-threatening autosomal recessive disease is characterized by progressive bacterial colonization in the lungs, ultimately leading to pulmonary failure. The absence or dysfunction of a CFTR protein in airway epithelium disrupts transepithelial ion transport, resulting in inadequate airway surface liquid (ASL) hydration and a thickened mucous layer, which impair mucociliary clearance and compromise innate immunity [3,4]. Although CFTR modulator therapies have significantly improved clinical outcomes, approximately 10% of CF patients, either those who produce minimal to no CFTR or who cannot tolerate modulators, continue to rely on symptomatic treatments [5]. Ongoing research aims to develop gene therapy approaches capable of restoring CFTR function in the lungs of all individuals with CF, regardless of their CFTR genotype [6]. Given that chronic infection in CF lungs is the primary cause of mortality in CF, gene therapy targeting CF lung disease remains a promising path toward a cure [7,8].

Despite the success of gene therapy in certain genetic diseases, clinical trials for CF lung disease have not yet achieved the desired outcomes [9,10]. This is partly due to the complex pathophysiology of CF lung disease and the heterogeneous pattern of *CFTR* expression across different cell types in the respiratory tract [11,12]. CFTR plays a pivotal role in coordinating ASL hydration, mucociliary clearance, and innate immune defense. In the airways, CFTR functions as an anion channel in a cell-type-specific manner and regulates or interacts with other ion channels, enabling fine-tuned control of airway surface fluid composition [13,14,15]. Unlike current FDA-approved gene therapy products that rely on gene addition for functional complementation, the ectopic expression of CFTR in CF lungs may not sufficiently restore airway homeostasis. Moreover, most luminal surface epithelial cells accessible to *CFTR* transfer vectors are terminally differentiated and have limited lifespans. Therefore, achieving durable CFTR expression would require periodic re-dosing throughout a patient’s lifetime [12,16].

The emergence of CRISPR (clustered regularly interspaced short palindromic repeats)-based genome editing has advanced gene therapy from functional complementation through gene addition to the precise correction of pathogenic mutations at the genomic level [17]. The CRISPR system comprises a CRISPR-associated protein (Cas), which is an RNA-guided DNA endonuclease, and a guide RNA (gRNA). Together, they form a ribonucleoprotein complex that recognizes a DNA target sequence through base-pair complementarity, enabling Cas to introduce a double-strand break (DSB) at a user-defined genomic site. Compared to *CFTR* addition, CRISPR-based *CFTR* editing offers distinct advantages for CF gene therapy—it has the potential to restore regulated *CFTR* expression under endogenous control in functionally relevant cell targets and may eliminate the need for repeat dosing if the airway stem/progenitor cells are effectively targeted.

CRISPR-induced DSBs are repaired through two major pathways—non-homologous end joining (NHEJ) and homology-directed repair (HDR) [18]. NHEJ is the primary repair mechanism throughout the cell cycle; it is rapid and active not only in dividing but also in non-dividing cells. In contrast, HDR occurs less frequently and is largely restricted to the dividing cells at the S and G2 phases. Nevertheless, introducing a DSB near a mutation can enhance HDR frequency, enabling precise correction using an exogenously supplied homologous donor template. Most current therapeutic genome editing approaches rely on HDR to accurately rewrite the mutations at defective gene loci. Although NHEJ is error-prone, NHEJ-based excision (NHEJ-ex) has shown therapeutic potential for removing pathogenic sequences or skipping defective exons to restore gene function, as demonstrated in the applications targeting the *Duchenne muscular dystrophy* (*DMD*) gene [19]. Another NHEJ-based editing strategy, homology-independent targeted integration (HITI), enables the insertion of therapeutic sequences into specific gene loci through a “cut-and-paste” mechanism to achieve a regulated expression under endogenous control, and has shown efficacy in post-mitotic cells both in vitro and in vivo [20]. In addition, new CRISPR technologies, such as base editing [21] and prime editing [22], enable precise nucleotide modifications without inducing DSBs and have begun to enter clinical trials.

To date, several CRISPR-based gene therapies are in clinical trials. CASGEVY^®^ is the first ex vivo genome editing treatment approved for use, utilizing autologous stem cells [23]. In the context of CF, CRISPR-based *CFTR* editing approaches have been explored in primary airway basal cells and induced pluripotent stem cells (iPSCs) from CF patients, as well as in CF animal models such as ferrets and pigs. Upon differentiation, these edited cells have demonstrated proof of functional restoration in either polarized airway epithelium or organoid cultures [24,25,26,27,28,29]. However, ex vivo therapies face significant challenges, including the need to expand a sufficient number of edited cells while preserving their capacity to differentiate into the diverse CFTR-expressing epithelial cell types, and the difficulty of effectively delivering these cells to the appropriate regions of the lung.

For in vivo application, the correction of *CFTR* mutations in terminally differentiated epithelial cells may restore functional CFTR expression for the remainder of each edited cell’s lifespan. A permanent cure, however, would require targeting airway progenitor cells capable of self-renewal and differentiation into multiple *CFTR*-expressing cell types [30,31]. Given that HDR is limited to dividing cells and most surface airway epithelial cells, including progenitors, are post-mitotic under homeostatic conditions [32], the HITI approach represents a promising strategy for in vivo *CFTR* editing in CF lungs. Basal cells, the primary progenitor population in the conducting airways, can be transduced by an airway-tropic recombinant adeno-associated viral vector, rAAV2.5T [33], in polarized human airway epithelium cultured at an air–liquid interface (HAE-ALI) [34]. This finding suggests that the airway progenitors could be targeted in vivo, although they are relatively less accessible beneath luminal columnar cells.

In this study, we first explored the HITI approach in primary airway basal cells isolated from the transgenic ROSA^mTmG^ ferret [35] using rAAV2.5T vectors. To support efforts to enhance editing efficiency, we also developed a versatile reporter cell line derived from CuFi-8, an immortalized human airway basal cell line transformed from a CF patient with a homozygous F508del genotype [36], enabling quantitative assessment of HDR- and NHEJ-based editing outcomes at the same genomic locus.

## 2. Materials and Methods

### 2.1. Lentiviral Vectors Production

Lentiviral vector transfer plasmid: pLentiCas9-Blast (Addgene plasmid #52962) expresses a self-cleaving fusion protein of SpCas9 and BSD (a product of the blasticidin S resistance gene) [37].

Lentiviral vector production: LentiCas9-Blast is a VSV-G pseudotyped lentiviral vector generated from the transfection of pLentiCas9-Blast alongside helper plasmids in HEK293T cells. Functional titers as transducing units (TU)/mL were quantified using TaqMan PCR quantification of viral genome integration following the infection of HEK293T, as previously described [38].

### 2.2. rAAV Vector Production

Plasmids for the production of rAAV expression vectors: pAAV2.Cre is an rAAV2 proviral transfer plasmid that encodes a Cre recombinase (Cre) driven by a CMV (Cytomegalovirus) immediate early promoter and enhancer [39]. pAAV2.EFsCas9, a gift from Ryohei Yasuda (Addgene plasmid # 104588), expresses spCas9 driven by the short form of the *human eukaryotic translation elongation factor 1 alpha* (*EF1α*) promoter (EFs-pro), the short *EF1α* promoter without the enhancer at intron1 [40].

Plasmids for the production of rAAV gene editing vectors: pAAV2.HITIhrGFP was used to produce rAAV2.5T.HITIhrGFP for the targeted insertion of a splice acceptor-associated hrGFP reporter sequence (referred to as the HITI donor) into the chimeric intron of the CAG promoter (CAGp). It contains the HITI donor flanked by gRNA CAG-g3 recognition sequences (tttatggtaatcgtgcg//agaGGG) in an inverted direction matching its orientation in the ferret genome to allow for the excision of the HITI donor from the rAAV transduction double-stranded (ds)DNA intermediates and the following HITI into the targeted site. The vector also carries a U6 promoter-driven CAG-g3 expression cassette. To prevent unwanted transcriptional read-through from the cryptic promoter within the AAV ITR or vector elements, an SV40 polyadenylation sequence was placed upstream of the HITI donor. pAAV2.HITI(5N) was designed as the HITI donor vector targeting the gene editing reporter (GER) cassette (see Section 2.3). The targeted site lies within the “5N” sequence “tatacgaagttatgtcg//actAGG”, which is located between the CBh promoter and the start codon of Y66S *eGFP* and recognized by the gRNA g5N. rAAV.HITI(5N) expresses g5N using the U6 promoter and carries the HITI donor sequence, flanked by oppositely oriented 5N sequences. An insulator sequence (Ins) was included upstream of the donor sequence to suppress potential transcriptional leakage by the cryptic promoter activity from AAV ITR and/or other elements in the vector genome. pAAV2.EXCI expresses spCas9 and the gRNA gSTOPout. It was constructed based on the pAAV2-EFsCas9; the EFs-pro for spCas9 expression was replaced by the 83 bp short synthetic promoter (tg83 pro), enabling the inclusion of the gSTOPout expression cassette. pAAV2.tempG551Y66-gRNA(2) was the previously validated Y66S eGFP editing vector. We had pseudotyped this vector in both AAV6 and AAV2.5T capsids to transduce the primary ferret airway basal cells, for the correction of the Y66S mutation in the non-fluorescent *eGFP* mutant [24]. In this study, we produced this HDR vector and termed it in short as rAAV2.5T.HDR(Y66). The gRNAs used for this study were chosen using the online tool CRISPOR (http://crispor.tefor.net, accessed on 15 May 2019). In vitro cleavage assays were performed as previously described to validate the potency of the chosen gRNAs [24]. Briefly, DNA fragments containing the gRNA recognition site were incubated with the ribonucleoprotein complexes formed by synthetic sgRNA and recombinant Cas9 proteins (Integrated DNA Technologies (IDT) Inc., Coralville, IA, USA). Following Proteinase K digestion, the reaction was resolved in agarose electrophoresis to visualize the cleavage of the DNA substrate. Supplementary Figure S1 shows an example of the validation of gRNAs targeting the intron of the CAG promoter.

rAAV vector production: rAAV vectors were packaged in the AAV2.5T capsid. The rAAV2.5T vectors were generated from the triple transfection of the rAAV2 proviral transfer plasmid with two helper plasmids pAd4.1 and pAAVRep2Cap6 or pAAVRep2Cap2.5T, as previously described [41]. TaqMan PCR determined the titers of these vectors as DNase I-resistant particles (DRP)/μL.

### 2.3. PiggyBac (PB) Transfer Plasmid and Transposase Expression Plasmid

The gene editing reporter cassette, “CBh(pro)-LoxP-Y66SeGFP-STOP-LoxP-mCherry” (hereafter referred to as the gene editing reporter, or GER), was cloned into a PiggyBac (PB) donor plasmid pBluescript-minimal PB (a gift from Dr. Mario Capecchi’s lab, the University of Utah School of Medicine, Addgene #177151) alongside a puromycin N-acetyltransferase (*pac*) gene as a selectable marker conferring puromycin resistance. Gene synthesis services were performed by Genscript Biotech Corp. (Piscataway, NJ, USA). The resulting PB donor plasmid was named pPB-GERpuro. The PB transposase expression plasmid, pSuperPBTase, was obtained from System Biosciences (PB210PA-1, Palo Alto, CA, USA).

### 2.4. Cell Cultures

Proliferating cultures: CuFi-8 cells and primary ferret airway epithelial cells were cultured and passaged in PneumaCult^TM^-Ex Plus medium (StemCell Technologies, Vancouver, BC, Canada) on plastic plates or dishes pre-coated with collagen IV (Sigma, St. Louis, MO, USA).

Polarized epithelial cultures: 1.5 × 10^5^ CuFi-8 cells or their derivative GER cells or primary ferret basal cells were seeded onto 6.5 mm, 0.4 μm pore size, polyester Transwell^®^ inserts (Costar^®^#3470, Corning Inc., Kennebunk, ME, USA) that were precoated with collagen IV. Seeding occurred in PneumaCult^TM^-Ex Plus medium (StemCell Technologies). At 24 h post seeding, the media in both apical and basal chambers were replaced with PneumaCult^TM^ ALI medium (StemCell Technologies). The cultures were then air-lifted the following day to facilitate differentiation at ALI for three weeks [42]. The medium in the basal chamber was replenished every other day in the first week, then twice a week during the culturing period. Matured polarized airway epithelium cultures with a transepithelial electrical resistance (TEER) >1000 Ω/cm^2^ were used for experiments [43].

### 2.5. Lentiviral Vector Transduction

Cells were seeded onto a well of 6-well plate at the density of 2 × 10^5^ cells per well. The day after seeding, the cells were transduced with LentiCas9-Blast at a multiplicity of infection (MOI) of 1.5 TU/cell. Selection for puromycin (Puro)-resistant cells was started two days after lentiviral infection in PneumaCult^TM^-Ex Plus medium supplemented with 1 μg/mL Puro. In this condition, all mock-infected control cells died within 2 days after exposure to Puro, allowing for the rapid selection of a Puro-resistant polyclonal pool within a week.

### 2.6. rAAV Vector Transduction

Proliferating airway basal cells were seeded into 6-well plates at a density of 3.3 × 10^5^ cells/well in PneumaCult^TM^-Ex Plus medium. After overnight incubation, rAAV transductions were conducted. The inoculum was removed after 6 h of incubation.

For the transduction of polarized HAE cultures, rAAV vectors were diluted in 100 μL PneumaCult^TM^-ALI medium and applied to the apical chamber. Because rAAV apical transduction encounters post-entry barriers that limit nuclear transport [44,45], doxorubicin (Dox) was used to enhance rAAV2.5T transduction efficiency. Dox (2.5 µM) was added to the basal chamber medium during the transduction period (16 h), facilitating rAAV nuclear transport and promoting productive transduction in polarized HAE-ALI [46]. After transduction, the inoculum was aspirated from the apical chamber, and the medium in the basal chamber was replaced with fresh PneumaCult^TM^-ALI medium without Dox.

### 2.7. Flow Cytometry Analysis

rAAV-transduced cells were harvested using Accutase (Innovative Cell Technologies, Inc., San Diego, CA, USA) and centrifuged at 1000 rpm for 5 min. Cell pellets were washed with phosphate-buffered saline (PBS) and passed through a 70 µm cell strainer to obtain a single-cell suspension. The suspension was centrifuged at 1000 rpm for 5 min and resuspended in PBS. Cell concentration was adjusted to 1.0 × 10^6^ cells/mL. Non-infected cells were used as a control. Flow cytometry was performed using a BD LSR II flow cytometer (Becton Dickinson, San Jose, CA, USA), equipped with 405 nm, 488 nm, 561 nm, and 639 nm lasers. mCherry was excited using a 561 nm laser, and emission was detected with a 610/20 bandpass filter. eGFP was excited with a 488 nm laser and detected using a 530/30 bandpass filter. Data acquisition was performed using FACSDiva software v9.0 (Becton Dickinson).

## 3. Results

### 3.1. Homology-Independent Targeted Insertion in Primary Ferret Airway Epithelial Cultures

**Figure 1:**
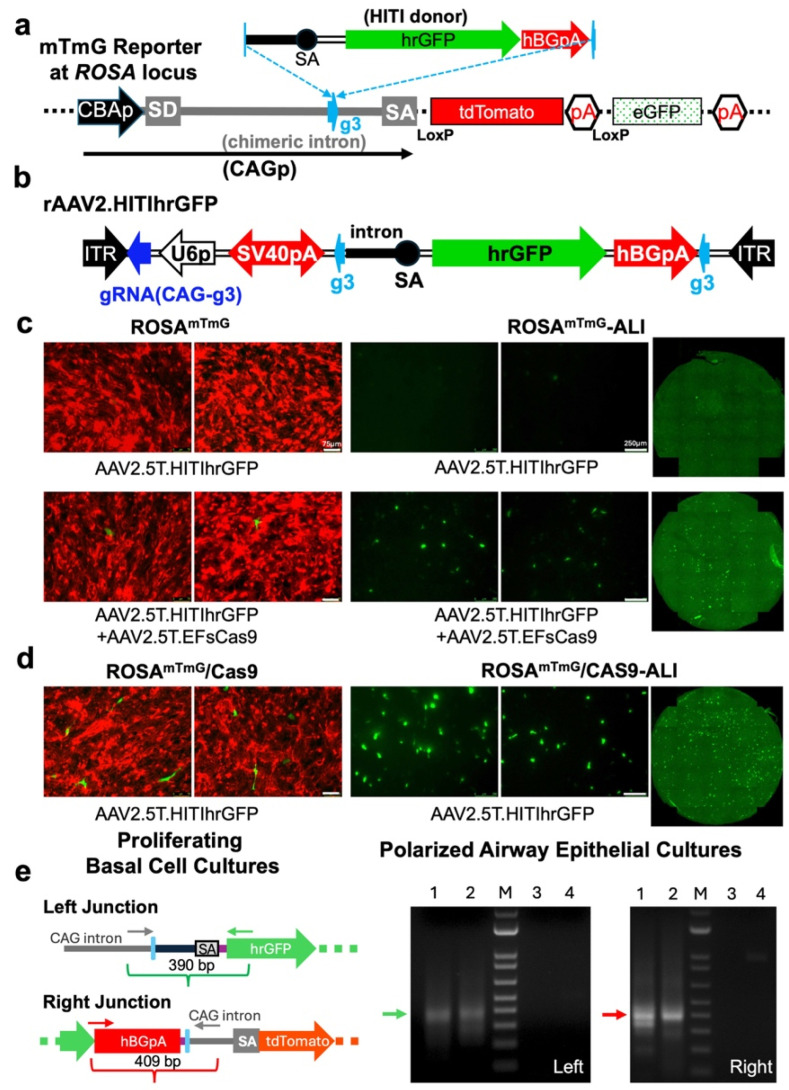
Homology-independent targeted integration (HITI) in primary ferret epithelial cultures. (**a**) A schematic of the HITI donor design and its targeted insertion into the chimeric intron of the CAG promoter (CAGp) using an exon trap strategy to drive the expression of the promoterless hrGFP reporter. The gRNA CAG-g3 targets the g3 sequence within the chimeric intron. (**b**) A map of the rAAV HITI donor vector. (**c**,**d**) HITI-mediated hrGFP expression in airway epithelial cultures derived from the tracheobronchial cells isolated from ROSA^mTmG^ ferrets (**c**) or in cultures stably expressing Cas9 (**d**). The proliferating basal cell cultures or well-differentiated ALI cultures were transduced with the HITI donor vector alone at a multiplicity of infection (MOI) of 50K or co-transduced with a Cas9-expressing vector (MOI of 50K each). The images were captured 8 days post transduction. Representative fluorescence images are shown, including tile-scanned images of ALI cultures on the Transwell^®^ (Costar^®^#3470) membrane. The red fluorescence channel is omitted in the ALI culture images for clarity. (**e**) PCR confirmation of targeted HITI at the g3 site. Primer sets spanning the genome–insert junctions were used—one primer located in the CAG intron (grey) and the other within the HITI donor sequence (colored)—amplifying both the upstream (left, green arrow points) and downstream (right, red arrow points) junctions. Lane 1: Dual-AAV transduction in ROSA^mTmG^ cells; Lane 2: HITI vector alone in ROSA^mTmG^/Cas9 cells; Lane 3: Blank; Lane 4: HITI donor vector alone in ROSA^mTmG^ cells; M: Invitrogen DNA 1 kb Plus Ladder (bands shown from 100 bp to 2.0 kb).

To assess the feasibility of HITI in ferret airway epithelial cells, we used primary basal cells isolated from a transgenic ROSA^mTmG^ ferret. This reporter line was generated by integrating a Cre-recombinase (Cre)-responsive mTmG cassette [CAGp-(LoxP-tdTomato-STOP-LoxP-eGFP)] into the *ROSA26* locus, a genomic safe harbor [35]. In the absence of Cre, ROSA^mTmG^ ferret constitutively expresses tdTomato under the control of the CAG promoter (CAGp) [47]. To test a targeted exon trap strategy using HITI, we designed a donor sequence carrying a splice acceptor-linked hrGFP reporter (referred to as the HITI donor) and targeted it to the chimeric intron of CAGp. Successful HITI at the gRNA CAG-g3 recognition sequence (g3), located 306 bp upstream of the splice acceptor–exon junction, was expected to redirect transcriptional output from *tdTomato* to *hrGFP*, resulting in red-to-green fluorescence conversion in the edited cells. (Figure 1a). The rAAV2.HITIhrGFP served as the HITI donor vector, carrying both the HITI donor and a U6 promoter-driven CAG-g3 expression cassette (Figure 1b). The HITI donor is flanked by CAG-g3 recognition sites to enable its excision from dsDNA intermediates formed during rAAV transduction from the single-stranded genome.

Ferret airway basal cells can be enriched and expanded from primary epithelial cells obtained from tracheal and bronchial tissues. These cells can differentiate into polarized epithelium when cultured at an ALI [24]. Basal cells isolated from ROSA^mTmG^ ferrets also retain this differentiation potential (Supplementary Figure S2). We tested rAAV2.5T-mediated HITI in proliferating basal cultures and polarized ferret airway epithelial ALI cultures (FAE-ALI) derived from ROSA^mTmG^ ferrets (Figure 1c). In both conditions, only a few hrGFP-positive cells were detected following co-transduction with rAAV2.5T.HITIhrGFP and the Cas9 expression vector, rAAV2.5.EFsCas9, at an MOI of 5 × 10^4^ DRP/cell (5OK) each. No green fluorescent cells were observed in the cultures transduced with the donor vector alone.

In the co-transduction experiment using rAAV2.5T.eGFP and rAAV2.5T.mCherry in CuFi-ALI cultures, we detected 49.9 ± 6.4% eGFP-positive cells and 56.4 ± 6.47% mCherry-positive cells, with 42.0 ± 6.9% of the cells expressing both reporters (see Supplementary Figure S3). These results suggest a high rate of dual-AAV co-delivery to the same recipient cells. Therefore, we hypothesized that Cas9 expression may be a rate-limiting factor, as the observed low editing efficiencies were unlikely due to inefficient vector co-delivery. To test this, we established a stable Cas9-expressing ROSA^mTmG^ basal cell line (referred to as ROSA^mTmG^/Cas9 cells) via lentiviral transduction with LentiCas9-Blast, followed by blasticidin S (Blast) selection. Both proliferating basal cells and polarized FAE-ALI cultures derived from ROSA^mTmG^/Cas9 cells were transduced with rAAV2.5T.HITIhrGFP alone (Figure 1d). Compared to the original co-transduction strategy, more hrGFP-positive cells were observed in the test groups that did not rely on dual-AAV delivery. However, overall HITI efficiency remained low under all tested conditions. Targeted *hrGFP* insertion was confirmed by PCR amplification of both the 5′ and 3′ junctions, using the primer sets with one primer anchored at the CAG intron and the other located within the HITI donor sequence (Figure 1e).

### 3.2. Generation of Gene Editing Reporter Cell Line Derived from CuFi-8 Cells

Although the exon trap HITI strategy demonstrated proof of concept in both proliferating basal cell cultures and well-differentiated ferret airway epithelial cultures, the overall editing efficiency remained low. Given that the differentiation capacity of primary basal cell cultures can decline with passage, we hypothesized that the immortalized CuFi-8 cell line could be engineered to establish a reproducible and easily assessable reporter system. Such a system would enable the optimization and advancement of gene editing techniques. CuFi-8 cells also maintain a basal cell phenotype in proliferating cultures and can differentiate into pseudostratified mucociliary epithelium when cultured at an ALI. These ALI cultures contain diverse epithelial cell types, including ciliated cells, secretory cells (goblet cells and club cells), basal stem cells, and a rare population of ionocytes [48]. Importantly, genetically modified CuFi-8 derivatives retain their differentiation capacity, making them a valuable model for studying airway biology and respiratory viral infection. Previously, CuFi-8 derivatives have been used to study rAAV2.5T airway transduction biology [49] and to investigate host–virus interactions during human bocavirus 1 infections in human airway epithelial cultures in vitro [50].

**Figure 2:**
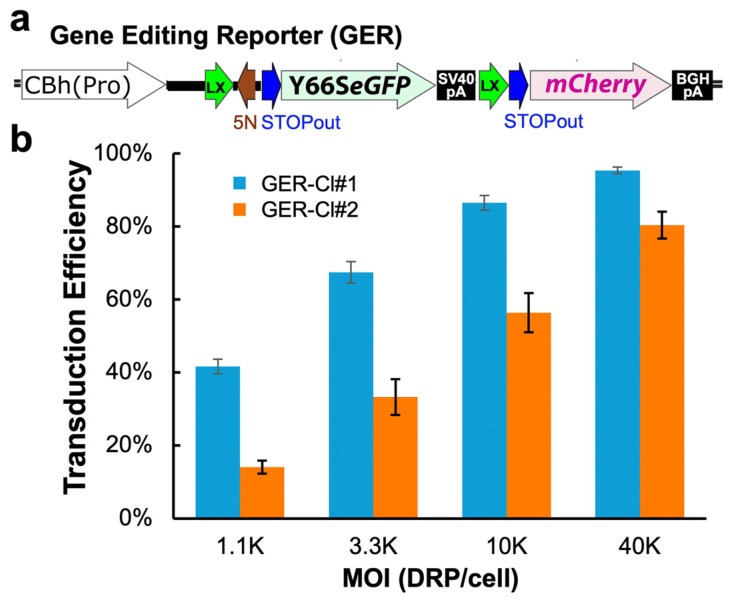
Cre recombinase-responsive mCherry expression in the CuFi-GER cells. (**a**) A schematic of the gene editing reporter (GER) cassette, which was stably integrated into CuFi-8 cells using the PiggyBac transposon system. Lx: LoxP site; 5N: sequence recognized by gRNA g5N; STOPout: sequence recognized by gRNA gSTOPout. (**b**) The dose-dependent activation of mCherry expression in two CuFi-GER lines, GER-Cl#1 and GER-Cl#2. The cells were transduced with rAAV2.5T.Cre at the indicated multiplicities of infection (MOIs). Three days post transduction, the percentage of the mCherry-positive cells was quantified by flow cytometry to assess the transduction efficiency. The data represent the mean ± standard error of the mean (SEM) from *n* = 6 independent transductions.

The GER cassette, “CBh(pro)-LoxP-Y66SeGFP-STOP-LoxP-mCherry” (Figure 2a), was designed to enable the generation of gene editing reporter cell lines. It expresses a non-fluorescent mutant of eGFP (Y66S eGFP) [51] under the control of the CBh promoter [CBh(pro)] [52], enabling assessment of HDR-mediated repair through the real-time visualization of green fluorescence restoration. The cassette also includes a “LoxP-STOP-LoxP” (LSL) element upstream of *mCherry,* conferring Cre-responsive mCherry expression. This feature allows for the quantification of Cre-mediated recombination as a proxy for evaluating the delivery efficiency of rAAV or other viral and non-viral vectors. Additionally, mCherry expression driven by the CBh(pro) can be activated independently of Cre through the CRISPR/Cas9-mediated excision of the LSL element, followed by the NHEJ of the resulting DSBs.

The GER cassette was cloned into a PiggyBac (PB) donor plasmid together with a puromycin N-acetyltransferase (*pac*) gene to generate the PB donor plasmid pPB-GERpuro. CuFi-GER cells were produced by co-transfection of the pPB-GERpuro and pSuperPBTase, followed by Puro selection. Six independent CuFi-GER cell lines were obtained through single-cell expansion. All clones exhibited mCherry expression following rAAV2.5T.Cre transduction, confirming the successful integration of the GER cassette. Since the parental CuFi-8 cell line is heterogeneous and PiggyBac transposase-mediated integration is random to a TTAA tetranucleotide site, these clones are considered distinct. They exhibited variable propagation rates during passaging, with two showing limited proliferative capacity.

We selected two of these lines, GER-Cl#1 and GER-Cl#2, for further experiments based on their robust and comparable growth characteristics. TaqMan PCR analysis revealed ~2 integrated GER copies per diploid genome in both lines, normalized to the *CFTR* copies [*eGFP*: *CFTR* = 1.18 ± 0.28 (GER-Cl#1) and 0.98 ± 0.20 (GER-Cl#2)]. When transduced with rAAV2.5T-Cre at varying MOIs, both lines displayed distinct dose-dependent mCherry reporter expressions (Figure 2b). GER-Cl#1 was more susceptible to rAAV2.5T transduction, achieving >80% mCherry-positive cells at an MOI of 10K. In contrast, GER-Cl#2 required an MOI greater than 40K to reach a comparable level of reporter expression. This discrepancy may reflect differences in genomic integration sites, and/or genetic and epigenetic variability among clones derived from the heterogeneous parental CuFi-8 population.

**Figure 3:**
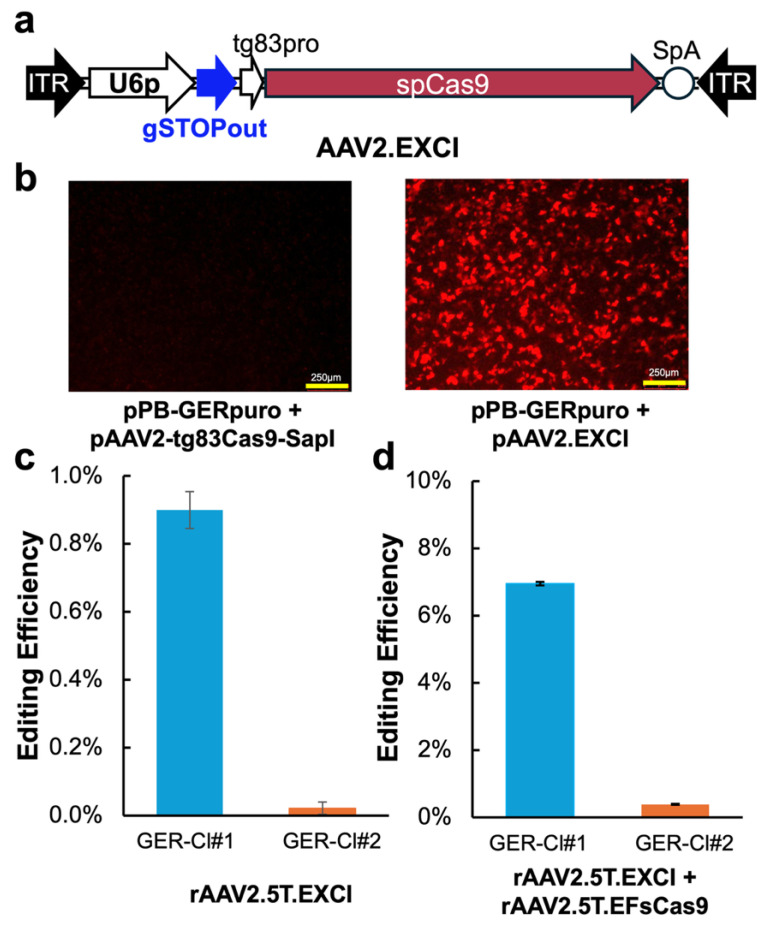
CRISPR-mediated sequence excision activated reporter expression in CuFi-GER cells. (**a**) A schematic of the gene editing rAAV vector designed for non-homologous end joining-based excision (NHEJ-ex). U6p: U6 promoter; tg83pro: 83 bp synthetic promoter. SpA: synthetic polyadenylation signal sequence. (**b**) The validation of the rAAV proviral transfer plasmid, pAAV2.EXCI, in HEK 293 cells. The cells were co-transfected with the plasmids as indicated. The fluorescence images were captured at 30 h post transfection. (**c**,**d**) The reporter cell lines, GER-Cl#1 and GER-Cl#2, were cultured on 6-well plates and transduced with rAAV2.5T.EXCI alone at MOIs of 50K and 100K, respectively, (**c**) and co-transduced with the Cas9 expression vector rAAV2.5T.EFsCas9 at an MOI of 50K for both lines (**d**). Six days post transduction, the percentage of the mCherry-positive cells was quantified by flow cytometry to assess NHEJ-ex editing efficiency. The data represent the mean ± standard deviation (SD) from *n* = 3 independent transductions.

To test the utility of these lines for reporting the gene editing outcomes, we first tested rAAV-mediated NHEJ-ex, in which CRISPR excises the LSL element from the GER cassette, resulting in mCherry expression. We identified a gRNA, designated gSTOPout, targeting the sequence “ggatccaccggtcgcca//ccaTGG”, which is present twice in the GER cassette, located in the 5′ untranslated region (5′ UTR) of both the Y66S *eGFP* and *mCherry* coding sequences. Initially, we aimed to deliver both spCas9 expression and the U6 promoter-driven gSTOPout using a single rAAV vector. To this end, we constructed a proviral transfer plasmid, pAAV2.EXCI, based on the pAAV2-EFsCas9. We incorporated the U6-gSTOPout expression cassette and replaced the EFs-pro with the compact 83 bp synthetic promoter tg83 [53] to drive spCas9 expression, allowing the total genome size to remain within the 5.0 kb packaging limit of rAAV (Figure 3a). The functionality of this construct was validated by co-transfecting HEK293 cells with pAAV2.EXCI and pPB-GERpuro; mCherry reporter expression was detectable within 30 h post transfection, confirming that both spCas9 and the gRNA were functionally expressed from pAAV2.EXCI (Figure 3b).

Next, we packaged rAAV2.5T.EXCI and transduced GER-Cl#1 at an MOI of 50K and GER-Cl#2 at an MOI of 100K. Despite the expected high transduction rates based on prior rAAV2.5T.Cre experiments (Figure 2b), flow cytometry revealed that only 0.9% of the GER-Cl#1 cells and 0.02% of the GER-Cl#2 cells expressed mCherry (Figure 3c). Increasing the MOI further impaired cell proliferation without improving editing efficiency. We suspected that the weak activity of the tg83 promoter in CuFi-8 cells may have resulted in insufficient Cas9 expression, leading to low editing outcomes.

To address this limitation, we implemented a dual-AAV vector strategy. GER-Cl#1 was co-transduced with rAAV2.5T.EXCI and the rAAV2.5T.EFsCas9 vector, each at MOI 50K. GER-Cl#2 received rAAV2.5T.EXCI at MOI 100K and rAAV2.5T.EFsCas9 at MOI 50K. This approach dramatically improved the editing outcomes. In the GER-Cl#1 cells, 6.95% of the cells were mCherry-positive, representing a 7.2-fold increase in NHEJ-ex efficiency. In the GER-Cl#2 cells, mCherry expression increased to 0.39%, reflecting an 18.2-fold improvement over rAAV2.5T.EXCI alone (Figure 3d). However, this fold change should be interpreted cautiously given the very low baseline editing observed in GER-Cl#2 with rAAV2.5T.EXCI alone.

### 3.3. Evaluation of Homology-Independent Targeted Integration in CuFi-GER Cell Lines

**Figure 4:**
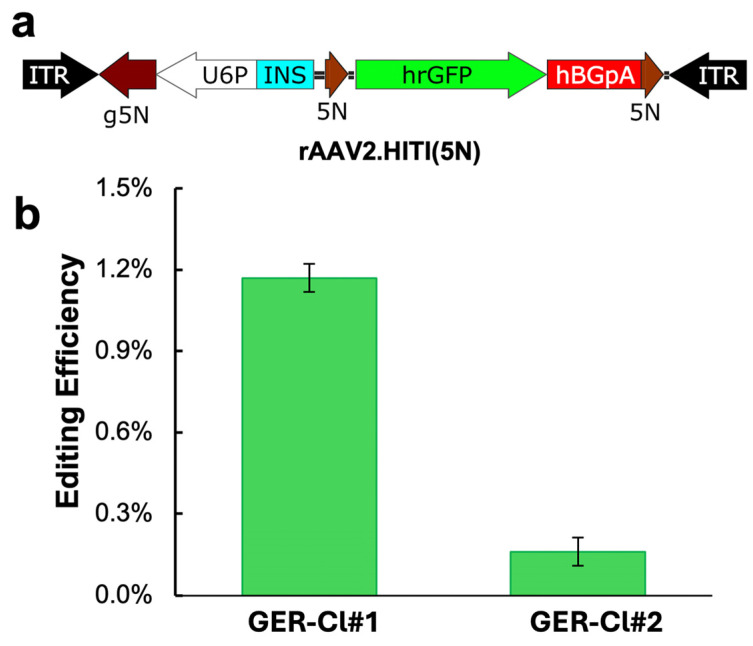
The homology-independent targeted insertion (HITI) of an hrGFP reporter into the GER cassette in CuFi-GER cells. (**a**) A schematic of the HITI donor vector. ITR: (AAV) inverted terminal repeat sequence. 5N: gRNA 5N recognition sequence. INS: transcriptional insulator sequence. hBGpA: human ß-globin polyadenylation signal sequence. (**b**) The reporter cell lines (GER-Cl#1 and GER-Cl#2) were cultured in 6-well plates and co-transduced with rAAV2.5T.HITI(5N) at MOIs of 50K and 100K, respectively, and the Cas9 expression vector rAAV2.5T.EFsCas9 at an MOI of 50K for both lines. Six days post transduction, the percentage of hrGFP-positive cells was quantified by flow cytometry to assess the HITI editing efficiency. The data represent the mean ± standard deviation (SD) from *n* = 3 independent transductions.

We next evaluated the HITI approach in CuFi-GER cell lines using an exon trap strategy similar to that previously tested in airway basal cells from ROSA^mTmG^ ferrets (Figure 1). A new HITI donor vector, rAAV2-HITI(5N), was constructed (Figure 4a). The vector carries a promoterless hrGFP coding sequence and expresses a U6 promoter-driven gRNA g5N, which targets the 5N sequence located upstream of the Y66S *eGFP* coding sequence within the GER cassette. The rAAV genome was packaged into an AAV2.5T capsid.

The GER-Cl#1 and GER-Cl#2 cells were co-transduced with rAAV2.5T.HITI(5N) and rAAV2.5T.EFsCas9. Editing efficiencies were quantified by flow cytometry based on the percentage of hrGFP-positive cells. The results showed HITI efficiencies of 1.17% in GER-Cl#1 and 0.16% in GER-Cl#2 (Figure 4b). These outcomes indicate that HITI-mediated editing was less efficient than NHEJ-ex in both cell lines.

### 3.4. A Versatile Reporter Cell Line for Assessing NEHJ- and HDR-Based Gene Editing Efficiencies

**Figure 5:**
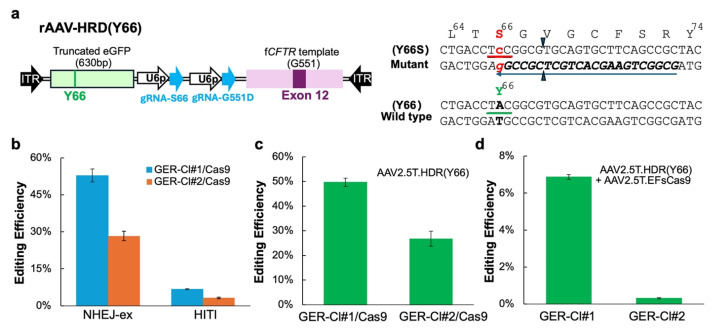
Gene editing in Cas9-expressing CuFi-GER cell lines. (**a**) A schematic of the HDR vector (left) and a portion of the e*GFP* coding sequence spanning from L64 to Y74 (mutant and wild type). ITR: AAV inverted terminal repeat sequence; U6p: U6 promoter. The light green box indicates the HDR template for Y66S correction, consisting of a truncated e*GFP* coding sequence (630 bp) containing the Y66 codon. The light pink box represents the HDR template for correcting the G551D mutation in f*CFTR*, comprising a segment of the ferret *CFTR* sequence containing exon 12. The gRNA-S66 recognition sequence in the e*GFP* coding sequence is highlighted by a dark blue arrow (reverse orientation). This gRNA specifically recognizes the mutant e*GFP* sequence, encompassing the Y66S codon but not the wild-type sequence. (**b**). A comparison of the editing efficiencies for two nonhomologous end joining (NHEJ)-based editing approaches, NHEJ-ex and HITI, in GER-Cl#1/Cas9 and GER-Cl#2/Cas9 cells. The cells were transduced with either rAAV2.5T.EXCI (NHEJ-ex) or rAAV2.5T.HITI(5N) (HITI). Six days post transduction, the editing efficiencies were quantified by flow cytometry based on the percentages of mCherry-positive cells (NHEJ-ex) or hrGFP-positive cells (HITI). (**c**,**d**) The evaluation of homology-directed repair (HDR) using the Y66S eGFP correction assay. (**c**) The GER-Cl#1/Cas9 and GER-Cl#2/Cas9 cells were transduced with rAAV2.5T.HDR(Y66) alone at MOIs of 50K and 100K, respectively. (**d**) The parental GER-Cl#1 and GER-Cl#2 cells were co-transduced with rAAV2.5T.HDR(Y66) at MOIs of 50K and 100K, respectively, and rAAV2.5T.EFsCas9 at an MOI of 50K for both lines. Six days post transduction, the eGFP-positive cells were quantified by flow cytometry to determine the HDR-editing efficiencies. The data represent the mean ± standard deviation (SD) from *n* = 3 independent transductions.

To enhance the utility and streamline the application of the GER system, we generated Cas9-expressing derivatives, the GER-Cl#1 and GER-Cl#2 lines, by lentiviral transduction using lentiCas9-Blast, followed by Blast selection. The resulting Blast-resistant cell pools were designated GER-Cl#1/Cas9 and GER-Cl#2/Cas9, respectively. We first re-evaluated the NHEJ-ex in these Cas9-expressing cells using AAV2/2.5T.EXCI alone. Flow cytometry revealed a 7.6-fold and 73.3-fold increase in the percentage of mCherry-positive cells in GER-Cl#1/Cas9 and GER-Cl#2/Cas9, respectively, compared to the corresponding parental lines (Figure 5b, NHEJ-ex; cf. Figure 3d).

We next tested the HITI in these Cas9-expressing cells using rAAV2.5T.HITI(5N) alone. Compared to the dual-AAV approach used in parental lines, HITI efficiencies improved by 5.7-fold and 19.9-fold in GER-Cl#1/Cas9 and GER-Cl#2/Cas9, respectively (Figure 5b, HITI cf. Figure 4b). Despite these anticipated improvements, HITI remained less efficient than NHEJ-ex in both cell lines.

To evaluate the HDR-mediated correction of the Y66S mutation in the eGFP reporter, we transduced the GER-Cl#1/Cas9 and GER-Cl#2/Cas9 cells with rAAV2.5T.HDR(Y66) alone. As comparative controls, the parental cell lines (GER-Cl#1 and GER-Cl#2) were co-transduced with rAAV2.5T.HDR(Y66) (Figure 5a) and rAAV2.5T.EFsCas9. The restoration of green fluorescence served as a readout of HDR efficiency and was quantified by flow cytometry. HDR editing was markedly enhanced in Cas9-expressing reporter cells, with GER-Cl#1/Cas9 and GER-Cl#2/Cas9 showing 7.2-fold and 86.0-fold increases, respectively, compared to their dual-AAV-transduced parental counterparts (Figure 5c,d).

Interestingly, although NEHJ is generally considered more efficient than HDR in mammalian cells, comparable editing efficiencies for NHEJ-ex and HDR were observed in Cas9-expressing lines: 52.83% (NHEJ-ex) vs. 49.64% (HITI-ex) in the GER-Cl#1/Cas9 cells, and 28.3% vs. 26.7% in GER-Cl#2/Cas9, respectively. A similar trend was observed in the dual-AAV-transduced parental lines. In the GER-Cl#1 cells, NHEJ-ex efficiency was 6.72% and HDR efficiency was 6.87%, while in the GER-Cl#2 cells, the NHEJ-ex efficiency was 0.39% and the HDR efficiency was 0.31% (Figure 5d vs. Figure 3d).

### 3.5. Gene Editing in Well-Differentiated Polarized Airway Epithelial Cultures Derived from the CuFi-GER Cell Line

To extend our study to a more physiologically relevant model, we evaluated HITI- and HDR-based editing in well-differentiated, polarized airway epithelial cultures derived from the Cas9-expressing GER cells. GER-Cl#1/Cas9 and GER-Cl#2/Cas9 cells were seeded onto Transwell^®^ inserts and cultured at an ALI using PneumaCult^TM^-ALI medium (StemCell Technologies) for three weeks to promote epithelial differentiation. ALI cultures derived from the GER-Cl#2/Cas9 cells developed robust epithelial barriers, as evidenced by TEER values exceeding 1000 Ω/cm². In contrast, the GER-Cl#1/Cas9 cultures showed poor differentiation, with apical medium leakage and low TEER values (<300 Ω/cm²), indicating compromised barrier integrity. These findings suggest that the GER-Cl#1/Cas9 cells may have lost their differentiation capacity; thus, subsequent gene editing experiments were conducted in GER-Cl#2/Cas9-ALI cultures.

**Figure 6:**
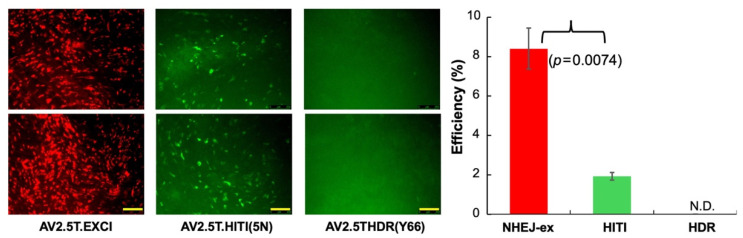
Gene editing in well-differentiated epithelium cultured at the air–liquid interface (ALI) derived from the GER-Cl#2/Cas cell lines. ALI cultures of GER-Cl#2/Cas9 cells were apically transduced with the indicated rAAV gene editing vectors. The fluorescence images were captured 6 days post transduction prior to flow cytometry analysis. Representative images are shown. Editing efficiencies were quantified by flow cytometry based on the percentage of mCherry-positive cells (NHEJ-ex) and hrGFP-positive cells (HITI). The data represent the mean ± standard deviation (SD) from *n* = 4 independent transductions. Statistical significance was analyzed using Student’s *t*-test.

Fully differentiated GER-Cl#2/Cas9-ALI cultures were apically transduced with three editing vectors: rAAV2/2.5T.EXCI, rAV2.5T.HITI(5N), and rAAV2.5T.HDR(Y66), respectively. Editing efficiency was quantified by flow cytometry. Transduction with rAAV2/2.5T.EXCI yielded 8.4% of cells being mCherry-positive, while transduction with rAV2.5T.HITI(5N) resulted in 1.9% of cells being hrGFP-positive, indicating that HITI is less efficient than NHEJ-ex (Figure 6), which is consistent with the observations in proliferating basal cells. As expected, no eGFP-positive cells were detected in the ALI cultures following rAAV2/2.5T.HDR(Y66) transduction, which is consistent with the known inefficiency of HDR in mitotically quiescent epithelial cells.

## 4. Discussion

Gene editing holds promise for permanently correcting defective genes while preserving their endogenous regulatory context, making it a compelling strategy for CF gene therapy with distinct advantages over traditional *CFTR* replacement strategies. Several CRISPR-based editing approaches are currently under investigation. Precise correction of specific *CFTR* mutations can be achieved through HDR, base editing, or primer editing. While these genotype-specific approaches align with personalized medicine. Alternatively, HITI of a *CFTR* mega-exon into the intronic sequence via exon trap may provide a universal, genotype-independent approach to bypass mutations downstream of the integration site. For in vivo *CFTR* editing in the lungs of individuals with CF, effective gene therapy necessitates targeting either CFTR-expressing cells or the lung stem/progenitor cells, which sustain epithelial homeostasis and regenerate the CFTR-expressing cell populations. However, CFTR-expressing cells are typically post-mitotic, and airway stem/progenitor cells remain mitotically quiescent during normal homeostasis [12,54,55], limiting the applicability of HDR-based strategies in vivo. Despite this, HDR remains valuable for ex vivo applications involving autologous stem cells or iPSCs. Regardless of the strategy, achieving efficient editing in the appropriate target cells is a critical challenge.

In a previous study, we used HDR to correct the G551D mutation of *CFTR* in ferret airway basal cells. Next-generation sequencing (NGS) revealed that NHEJ-mediated mutagenesis (i.e., indel formation) at the target site occurred more frequently than HDR-based correction [24], reinforcing the predominance of the NHEJ pathway in repairing CRISPR-induced DSBs in proliferating airway basal cells, even in the presence of an HDR template. In the present study, we evaluated the efficiency of rAAV2.5T-mediated HITI using an exon trap strategy designed to integrate a promoterless reporter into the intron of the CAGp. We tested this strategy in both proliferating and well-differentiated epithelial cultures derived from the transgenic ROSA^mTmG^ ferrets that carry the CAGp-mTmG cassette. As a proof of concept, HITI-mediated hrGFP expression was detected in both dividing and non-dividing epithelial cultures, indicating editing activity across cell states. However, overall editing efficiencies were low, highlighting the need for the further optimization of HITI-based strategies. Future efforts may benefit from the pharmaceutical modulation of DNA repair processes, as several small molecules that can enhance or inhibit NHEJ and HDR pathways have been identified [56,57,58].

To facilitate the application of such optimization efforts, we developed CuFi-GER cell lines capable of reporting HDR- and NHEJ-based editing events from a shared reporter cassette integrated at the same locus. This versatile system allows for the direct, side-by-side evaluation of the editing outcomes from NHEJ- and HDR-based approaches in a consistent cellular background, with the rapid quantification of editing efficiency via fluorescent reporter expression. The CuFi-GER system thus has the potential to be a platform for high-throughput or semi-high-throughput combinatorial screening for chemical and/or genetic agents to enhance gene editing efficiency. In this report, we evaluated NEHJ-ex, HITI, and HDR editing strategies in both proliferating basal cell cultures and well-differentiated epithelial cultures, using rAAV vectors for delivery. Several key observations from this reporter system were noteworthy:

First, although both NHEJ-ex and HITI rely on the NHEJ pathway, the editing efficiency of rAAV-mediated NHEJ-ex was approximately fivefold higher than that of HITI. This discrepancy likely reflects the greater molecular complexity required for HITI-mediated integration, which involves cleavages in both the target site and the donor vector, intermolecular ligation in trans, the correct orientation of the inserted sequence, and successful splicing into the host transcript. In contrast, NHEJ-ex requires only efficient cleavage and ligation at two defined sites. While this explanation is generally proper, editing efficiency can also be influenced by additional factors such as cutting efficiency, chromatin accessibility, and local sequence context, which are gRNA-dependent and may affect outcomes irrespective of editing strategy. For the NHEJ-excision strategy, we utilized the gSTOPout, which is present in two locations flanking the LSL-Y66SeGFP cassette, enabling excision using a single gRNA. However, this gRNA lies adjacent to the start codons of both Y66S-eGFP and mCherry and overlaps their Kozak consensus sequences, making it unsuitable for testing the HITI at this site. Future studies using matched target sites and standardized donor designs will be crucial for enabling more direct and controlled comparisons between insertion- and excision-based editing strategies.

Second, despite the widely accepted predominance of NHEJ over HDR in repairing DNA lesions in most cell types, we observed comparable efficiencies between NHEJ-ex-mediated mCherry expression and the HDR-mediated correction of Y66S mutation in *eGFP*. This unexpected result may be due to suboptimal gRNA efficiency or the requirement for simultaneous cleavage at two sites to excise the LSL element upstream of the *mCherry* coding sequence. Moreover, the gSTOPout targets the 5′UTR of *eGFP* and *mCherry*, regions that include the critical Kozak sequences. Indels introduced by error-prone NHEJ in these regions might impact the translation initiation of *mCherry*, thereby reducing observable reporter expression.

Third, our results suggest that Cas9 expression is a more significant rate-limiting factor for gene editing in CuFi airway epithelial cells in vitro than vector co-delivery efficiency. Although the dual-AAV delivery system has proven effective for gene editing in murine lungs in vivo [59,60], editing efficiencies in CuFi-GER cells in vitro remained relatively low. For example, in the GER Cl#1 cells, we observed editing efficiencies of 6.87% (HDR), 6.72% (NHEJ-ex), and 1.2% (HITI). In contrast, GER Cl#1/Cas9 cells, which constitutively express Cas9 and eliminate the need for co-transduction, achieved markedly higher efficiencies: 49.6% (HDR), 52.8% (NHEJ-ex), and 6.7% (HITI). Furthermore, our previous observations of the high co-expression of eGFP and mCherry in polarized CuFi-8 ALI cultures co-transduced with two separate rAAV reporter vectors (Supplementary Figure S2) support the notion that vector co-delivery to the same recipient cells is not a major limiting factor. Our data underscore that efficient editing in CuFi-GER cells is highly dependent on robust Cas9 expression, which presents a critical barrier to effective in vivo editing using rAAV. The ~4.7 kb packaging limit of rAAV constrains the inclusion of a strong promoter to drive spCas9 expression alongside a Pol III-driven gRNA within a single vector. As such, compact and potent Cas endonucleases, such as saCas9 [61], Cas12a (Cpf1) [62], and their enhanced derivatives like EbCas12a [63], represent attractive alternatives that allow for the incorporation of strong promoters (e.g., CMV IE promoter) to boost expression levels. However, persistent Cas expression is not necessarily required for genome editing; indeed, prolonged expression may increase the risk of off-target effects and cytotoxicity [64]. Since rAAV genomes can persist as an episome in post-mitotic cells, self-limiting or self-inactivating Cas constructs are under development to mitigate these risks [65,66]. Alternatively, the non-viral delivery of Cas mRNA or protein using emerging technologies, such as lipid nanoparticles (LNPs), may offer transient yet potent Cas activity suitable for therapeutic editing in airway epithelium in vivo [67,68]. Additionally, as Cas proteins are of bacterial origin, they may elicit host immune responses [69]. Transient immunosuppression or engineering less immunogenic Cas variants may be necessary to prevent the immune clearance of edited cells.

Fourth, we observed substantial variability in editing outcomes between cell lines. Editing efficiencies differed markedly between the two clonal lines derived from the single-cell expansion of the CuFi-8 parental population. GER-Cl#1 exhibited higher editing efficiencies than GER-Cl#2, with fold increases of 18 (NHEJ-ex), 7.2 (HITI), and 22.0 (HDR), respectively. These differences may result from genomic context effects due to the random integration of the GER cassette or intrinsic differences in DNA repair activity and/or pathway preference. This variability likely reflects genetic or epigenetic divergence acquired during the derivation of these clonal lines from the heterogeneous parental population. If this hypothesis is correct, comparative transcriptome analyses between these clonal lines could yield valuable insights into the host factors that modulate editing efficiency and guide further improvements.

Notably, while CRISPR-Cas9 is designed to target specific sequences, unintended insertions at cryptic or spontaneous DSBs, especially near active genomic elements, such as promoters or enhancers, are possible. In this study, ectopic hrGFP expression was undetectable when the HITI donor vector was delivered alone, suggesting that insertion at spontaneous DSBs resulting in detectable expression was rare. However, this observation is insufficient to exclude off-target or silent integration events elsewhere in the genome. Furthermore, the GER cell lines used in this study carry approximately two copies of integrated cassettes, which may reside at different loci and are not necessarily edited simultaneously. While our current study used flow cytometry as a convenient and scalable proxy for editing events, this approach does not directly confirm the precise molecular nature or zygosity of the genome modification. Therefore, the incorporation of sequencing-based methods in future experiments will be essential to validate reporter performance and accurately quantify editing efficiency. Employing high-resolution methods, such as targeted locus amplification and whole-genome sequencing, will be necessary to comprehensively evaluate the genome-wide insertional profile and ensure the safety of HITI-based strategies.

## 5. Conclusions

The CuFi-GER system described in this study provides a versatile and tractable model for studying DNA repair pathway dynamics, Cas9 delivery constraints, and cell line (clonal)-specific variability in gene editing efficiency. Its further application in chemical and genetic screening efforts could enable the development of effective pharmacologic strategies to modulate DNA repair pathways and enhance editing efficacy. Ultimately, such optimization could facilitate the successful application of gene editing therapies for CF and other pulmonary diseases.

## Data Availability

The original contributions presented in this study are included in the article. Further inquiries can be directed to the corresponding author.

## Supplementary Materials

The following supporting information can be downloaded at: https://www.mdpi.com/article/10.3390/v17060821/s1, Figure S1: Validation of candidate gRNAs by in vitro cleavage assay; Figure S2: Immunofluorescent analyses of epithelial cell types in polarized ferret airway epithelium cultured at an air–liquid interface (ALI); Figure S3: Dual rAAV2.5T co-transduction in well-differentiated CuFi-8 epithelial cultures.

## Abbreviations

The following abbreviations are used in this manuscript:

rAAV	Recombinant adeno-associated viral vector
NHEJ	Non-homologous end joining or homology-directed repair
NHEJ-ex	NHEJ-based excision
HDR	Homology-directed repair
HITI	Homology-independent targeted insertion
GER	Gene editing reporter
CRISPR	Clustered regularly interspaced short palindromic repeats
eGFP	Enhanced green fluorescent protein (derived from the Aequorea victoria jellyfish)
hrGFP	Humanized renilla green fluorescent protein (from the Renilla reniformis species)
DSB	Double-stranded DNA break
Blast	Blasticidin S
Puro	Puromycin
PBS	Phosphate-buffered saline
MOI	Multiplicity of infection
TEER	Transepithelial electrical resistance
Cre	Cre recombinase

## References

[1] Riordan J.R., Rommens J.M., Kerem B., Alon N., Rozmahel R., Grzelczak Z., Zielenski J., Lok S., Plavsic N., Chou J.L. (1989). Identification of the cystic fibrosis gene: Cloning and characterization of complementary DNA. Science.

[2] Rommens J.M., Iannuzzi M.C., Kerem B., Drumm M.L., Melmer G., Dean M., Rozmahel R., Cole J.L., Kennedy D., Hidaka N. (1989). Identification of the cystic fibrosis gene: Chromosome walking and jumping. Science.

[3] Stoltz D.A., Meyerholz D.K., Welsh M.J. (2015). Origins of cystic fibrosis lung disease. N. Engl. J. Med..

[4] Elborn J.S. (2016). Cystic fibrosis. Lancet.

[5] Clancy J.P., Cotton C.U., Donaldson S.H., Solomon G.M., VanDevanter D.R., Boyle M.P., Gentzsch M., Nick J.A., Illek B., Wallenburg J.C. (2019). CFTR modulator theratyping: Current status, gaps and future directions. J. Cyst. Fibros..

[6] Graeber S.Y., Mall M.A. (2023). The future of cystic fibrosis treatment: From disease mechanisms to novel therapeutic approaches. Lancet.

[7] Choi S.H., Engelhardt J.F. (2021). Gene Therapy for Cystic Fibrosis: Lessons Learned and Paths Forward. Mol. Ther..

[8] Yan Z., McCray P.B., Engelhardt J.F. (2019). Advances in gene therapy for cystic fibrosis lung disease. Hum. Mol. Genet..

[9] Guggino W.B., Cebotaru L. (2020). Gene Therapy for Cystic Fibrosis Paved the Way for the Use of Adeno-Associated Virus in Gene Therapy. Hum. Gene Ther..

[10] Alton E., Armstrong D.K., Ashby D., Bayfield K.J., Bilton D., Bloomfield E.V., Boyd A.C., Brand J., Buchan R., Calcedo R. (2015). Repeated nebulisation of non-viral CFTR gene therapy in patients with cystic fibrosis: A randomised, double-blind, placebo-controlled, phase 2b trial. Lancet Respir. Med..

[11] Jiang Q., Engelhardt J.F. (1998). Cellular heterogeneity of CFTR expression and function in the lung: Implications for gene therapy of cystic fibrosis. Eur. J. Hum. Genet..

[12] Tang Y., Yan Z., Engelhardt J.F. (2020). Viral Vectors, Animal Models, and Cellular Targets for Gene Therapy of Cystic Fibrosis Lung Disease. Hum. Gene Ther..

[13] Lei L., Traore S., Romano Ibarra G.S., Karp P.H., Rehman T., Meyerholz D.K., Zabner J., Stoltz D.A., Sinn P.L., Welsh M.J. (2023). CFTR-rich ionocytes mediate chloride absorption across airway epithelia. J. Clin. Investig..

[14] Okuda K., Dang H., Kobayashi Y., Carraro G., Nakano S., Chen G., Kato T., Asakura T., Gilmore R.C., Morton L.C. (2021). Secretory Cells Dominate Airway CFTR Expression and Function in Human Airway Superficial Epithelia. Am. J. Respir. Crit. Care Med..

[15] Reihill J.A., Douglas L.E.J., Martin S.L. (2021). Modulation of Ion Transport to Restore Airway Hydration in Cystic Fibrosis. Genes.

[16] King N.E., Suzuki S., Barilla C., Hawkins F.J., Randell S.H., Reynolds S.D., Stripp B.R., Davis B.R. (2020). Correction of Airway Stem Cells: Genome Editing Approaches for the Treatment of Cystic Fibrosis. Hum. Gene Ther..

[17] Cox D.B., Platt R.J., Zhang F. (2015). Therapeutic genome editing: Prospects and challenges. Nat. Med..

[18] Ceccaldi R., Rondinelli B., D’Andrea A.D. (2016). Repair Pathway Choices and Consequences at the Double-Strand Break. Trends Cell Biol..

[19] Aslesh T., Erkut E., Yokota T. (2021). Restoration of dystrophin expression and correction of Duchenne muscular dystrophy by genome editing. Expert Opin. Biol. Ther..

[20] Suzuki K., Tsunekawa Y., Hernandez-Benitez R., Wu J., Zhu J., Kim E.J., Hatanaka F., Yamamoto M., Araoka T., Li Z. (2016). In vivo genome editing via CRISPR/Cas9 mediated homology-independent targeted integration. Nature.

[21] Rees H.A., Liu D.R. (2018). Base editing: Precision chemistry on the genome and transcriptome of living cells. Nat. Rev. Genet..

[22] Chen P.J., Liu D.R. (2023). Prime editing for precise and highly versatile genome manipulation. Nat. Rev. Genet..

[23] Healey N. (2024). Next-generation CRISPR-based gene-editing therapies tested in clinical trials. Nat. Med..

[24] Yan Z., Vorhies K., Feng Z., Park S.Y., Choi S.H., Zhang Y., Winter M., Sun X., Engelhardt J.F. (2022). Recombinant Adeno-Associated Virus-Mediated Editing of the G551D Cystic Fibrosis Transmembrane Conductance Regulator Mutation in Ferret Airway Basal Cells. Hum. Gene Ther..

[25] Suzuki S., Crane A.M., Anirudhan V., Barilla C., Matthias N., Randell S.H., Rab A., Sorscher E.J., Kerschner J.L., Yin S. (2020). Highly Efficient Gene Editing of Cystic Fibrosis Patient-Derived Airway Basal Cells Results in Functional CFTR Correction. Mol. Ther..

[26] Vaidyanathan S., Salahudeen A.A., Sellers Z.M., Bravo D.T., Choi S.S., Batish A., Le W., Baik R., de la O S., Kaushik M.P. (2020). High-Efficiency, Selection-free Gene Repair in Airway Stem Cells from Cystic Fibrosis Patients Rescues CFTR Function in Differentiated Epithelia. Cell Stem Cell.

[27] Krishnamurthy S., Traore S., Cooney A.L., Brommel C.M., Kulhankova K., Sinn P.L., Newby G.A., Liu D.R., McCray P.B. (2021). Functional correction of CFTR mutations in human airway epithelial cells using adenine base editors. Nucleic Acids Res..

[28] Bulcaen M., Kortleven P., Liu R.B., Maule G., Dreano E., Kelly M., Ensinck M.M., Thierie S., Smits M., Ciciani M. (2024). Prime editing functionally corrects cystic fibrosis-causing CFTR mutations in human organoids and airway epithelial cells. Cell Rep. Med..

[29] Zhou Z.P., Yang L.L., Cao H., Chen Z.R., Zhang Y., Wen X.Y., Hu J. (2019). In Vitro Validation of a CRISPR-Mediated CFTR Correction Strategy for Preclinical Translation in Pigs. Hum. Gene Ther..

[30] Rock J.R., Onaitis M.W., Rawlins E.L., Lu Y., Clark C.P., Xue Y., Randell S.H., Hogan B.L. (2009). Basal cells as stem cells of the mouse trachea and human airway epithelium. Proc. Natl. Acad. Sci. USA.

[31] Alysandratos K.D., Herriges M.J., Kotton D.N. (2021). Epithelial Stem and Progenitor Cells in Lung Repair and Regeneration. Annu. Rev. Physiol..

[32] Rawlins E.L., Hogan B.L. (2008). Ciliated epithelial cell lifespan in the mouse trachea and lung. Am. J. Physiol. Lung. Cell Mol. Physiol..

[33] Excoffon K.J., Koerber J.T., Dickey D.D., Murtha M., Keshavjee S., Kaspar B.K., Zabner J., Schaffer D.V. (2009). Directed evolution of adeno-associated virus to an infectious respiratory virus. Proc. Natl. Acad. Sci. USA.

[34] Cooney A.L., Brommel C.M., Traore S., Newby G.A., Liu D.R., McCray P.B., Sinn P.L. (2023). Reciprocal mutations of lung-tropic AAV capsids lead to improved transduction properties. Front. Genome Ed..

[35] Yu M., Sun X., Tyler S.R., Liang B., Swatek A.M., Lynch T.J., He N., Yuan F., Feng Z., Rotti P.G. (2019). Highly Efficient Transgenesis in Ferrets Using CRISPR/Cas9-Mediated Homology-Independent Insertion at the ROSA26 Locus. Sci. Rep..

[36] Zabner J., Karp P., Seiler M., Phillips S.L., Mitchell C.J., Saavedra M., Welsh M., Klingelhutz A.J. (2003). Development of cystic fibrosis and noncystic fibrosis airway cell lines. Am. J. Physiol. Lung Cell Mol. Physiol..

[37] Sanjana N.E., Shalem O., Zhang F. (2014). Improved vectors and genome-wide libraries for CRISPR screening. Nat. Methods.

[38] Choi S.H., Reeves R.E., Romano Ibarra G.S., Lynch T.J., Shahin W.S., Feng Z., Gasser G.N., Winter M.C., Evans T.I.A., Liu X. (2020). Detargeting Lentiviral-Mediated CFTR Expression in Airway Basal Cells Using miR-106b. Genes.

[39] Liu X., Luo M., Guo C., Yan Z., Wang Y., Lei-Butters D.C., Engelhardt J.F. (2009). Analysis of adeno-associated virus progenitor cell transduction in mouse lung. Mol. Ther..

[40] Nishiyama J., Mikuni T., Yasuda R. (2017). Virus-Mediated Genome Editing via Homology-Directed Repair in Mitotic and Postmitotic Cells in Mammalian Brain. Neuron.

[41] Yan Z., Lei-Butters D.C., Keiser N.W., Engelhardt J.F. (2013). Distinct transduction difference between adeno-associated virus type 1 and type 6 vectors in human polarized airway epithelia. Gene Ther..

[42] Yan Z., Sun X., Evans I.A., Tyler S.R., Song Y., Liu X., Sui H., Engelhardt J.F. (2013). Postentry processing of recombinant adeno-associated virus type 1 and transduction of the ferret lung are altered by a factor in airway secretions. Hum. Gene Ther..

[43] Zabner J., Smith J.J., Karp P.H., Widdicombe J.H., Welsh M.J. (1998). Loss of CFTR chloride channels alters salt absorption by cystic fibrosis airway epithelia in vitro. Mol. Cell.

[44] Duan D., Yue Y., Yan Z., Yang J., Engelhardt J.F. (2000). Endosomal processing limits gene transfer to polarized airway epithelia by adeno-associated virus. J. Clin. Investig..

[45] Yan Z., Lei-Butters D.C., Liu X., Zhang Y., Zhang L., Luo M., Zak R., Engelhardt J.F. (2006). Unique biologic properties of recombinant AAV1 transduction in polarized human airway epithelia. J. Biol. Chem..

[46] Hao S., Zhang X., Ning K., Feng Z., Park S.Y., Aksu Kuz C., McFarlin S., Richart D., Cheng F., Zhang E.Y. (2023). Identification of host essential factors for recombinant AAV transduction of the polarized human airway epithelium. J. Virol..

[47] Miyazaki J., Takaki S., Araki K., Tashiro F., Tominaga A., Takatsu K., Yamamura K. (1989). Expression vector system based on the chicken beta-actin promoter directs efficient production of interleukin-5. Gene.

[48] Park S.Y., Feng Z., Choi S.H., Zhang X., Tang Y., Gasser G.N., Richart D., Yuan F., Qiu J., Engelhardt J.F. (2025). Recombinant Adeno-Associated Virus Vector Mediated Gene Editing in Proliferating and Polarized Cultures of Human Airway Epithelial Cells. Hum. Gene Ther..

[49] Ning K., Kuz C.A., Cheng F., Feng Z., Yan Z., Qiu J. (2023). Adeno-Associated Virus Monoinfection Induces a DNA Damage Response and DNA Repair That Contributes to Viral DNA Replication. mBio.

[50] Ning K., Zhao J., Feng Z., Park S.Y., McFarlin S., Cheng F., Yan Z., Wang J., Qiu J. (2024). N6-methyladenosine modification of a parvovirus-encoded small noncoding RNA facilitates viral DNA replication through recruiting Y-family DNA polymerases. Proc. Natl. Acad. Sci. USA.

[51] Tran N.D., Liu X., Yan Z., Abbote D., Jiang Q., Kmiec E.B., Sigmund C.D., Engelhardt J.F. (2003). Efficiency of chimeraplast gene targeting by direct nuclear injection using a GFP recovery assay. Mol. Ther..

[52] Gray S.J., Foti S.B., Schwartz J.W., Bachaboina L., Taylor-Blake B., Coleman J., Ehlers M.D., Zylka M.J., McCown T.J., Samulski R.J. (2011). Optimizing promoters for recombinant adeno-associated virus-mediated gene expression in the peripheral and central nervous system using self-complementary vectors. Hum. Gene Ther..

[53] Zhang L.N., Karp P., Gerard C.J., Pastor E., Laux D., Munson K., Yan Z., Liu X., Godwin S., Thomas C.P. (2004). Dual therapeutic utility of proteasome modulating agents for pharmaco-gene therapy of the cystic fibrosis airway. Mol. Ther..

[54] Urban N., Cheung T.H. (2021). Stem cell quiescence: The challenging path to activation. Development.

[55] Wu M., Zhang X., Lin Y., Zeng Y. (2022). Roles of airway basal stem cells in lung homeostasis and regenerative medicine. Respir. Res..

[56] Yu C., Liu Y., Ma T., Liu K., Xu S., Zhang Y., Liu H., La Russa M., Xie M., Ding S. (2015). Small molecules enhance CRISPR genome editing in pluripotent stem cells. Cell Stem. Cell.

[57] Stack J.T., Rayner R.E., Nouri R., Suarez C.J., Kim S.H., Kanke K.L., Vetter T.A., Cormet-Boyaka E., Vaidyanathan S. (2024). DNA-PKcs inhibition improves sequential gene insertion of the full-length CFTR cDNA in airway stem cells. Mol. Ther. Nucleic Acids.

[58] Canny M.D., Moatti N., Wan L.C.K., Fradet-Turcotte A., Krasner D., Mateos-Gomez P.A., Zimmermann M., Orthwein A., Juang Y.C., Zhang W. (2018). Inhibition of 53BP1 favors homology-dependent DNA repair and increases CRISPR-Cas9 genome-editing efficiency. Nat. Biotechnol..

[59] Liang S.Q., Walkey C.J., Martinez A.E., Su Q., Dickinson M.E., Wang D., Lagor W.R., Heaney J.D., Gao G., Xue W. (2022). AAV5 delivery of CRISPR-Cas9 supports effective genome editing in mouse lung airway. Mol. Ther..

[60] Thomas S.P., Domm J.M., van Vloten J.P., Xu L., Vadivel A., Yates J.G.E., Pei Y., Ingrao J., van Lieshout L.P., Jackson S.R. (2023). A promoterless AAV6.2FF-based lung gene editing platform for the correction of surfactant protein B deficiency. Mol. Ther..

[61] Ran F.A., Cong L., Yan W.X., Scott D.A., Gootenberg J.S., Kriz A.J., Zetsche B., Shalem O., Wu X., Makarova K.S. (2015). In vivo genome editing using Staphylococcus aureus Cas9. Nature.

[62] Zetsche B., Gootenberg J.S., Abudayyeh O.O., Slaymaker I.M., Makarova K.S., Essletzbichler P., Volz S.E., Joung J., van der Oost J., Regev A. (2015). Cpf1 is a single RNA-guided endonuclease of a class 2 CRISPR-Cas system. Cell.

[63] Wang H., Zhou J., Lei J., Mo G., Wu Y., Liu H., Pang Z., Du M., Zhou Z., Paek C. (2024). Engineering of a compact, high-fidelity EbCas12a variant that can be packaged with its crRNA into an all-in-one AAV vector delivery system. PLoS Biol..

[64] Ling S., Yang S., Hu X., Yin D., Dai Y., Qian X., Wang D., Pan X., Hong J., Sun X. (2021). Lentiviral delivery of co-packaged Cas9 mRNA and a Vegfa-targeting guide RNA prevents wet age-related macular degeneration in mice. Nat. Biomed. Eng..

[65] Li A., Lee C.M., Hurley A.E., Jarrett K.E., De Giorgi M., Lu W., Balderrama K.S., Doerfler A.M., Deshmukh H., Ray A. (2019). A Self-Deleting AAV-CRISPR System for In Vivo Genome Editing. Mol. Ther. Methods Clin. Dev..

[66] Ibraheim R., Tai P.W.L., Mir A., Javeed N., Wang J., Rodriguez T.C., Namkung S., Nelson S., Khokhar E.S., Mintzer E. (2021). Self-inactivating, all-in-one AAV vectors for precision Cas9 genome editing via homology-directed repair in vivo. Nat. Commun..

[67] Wei T., Sun Y., Cheng Q., Chatterjee S., Traylor Z., Johnson L.T., Coquelin M.L., Wang J., Torres M.J., Lian X. (2023). Lung SORT LNPs enable precise homology-directed repair mediated CRISPR/Cas genome correction in cystic fibrosis models. Nat. Commun..

[68] Li B., Manan R.S., Liang S.Q., Gordon A., Jiang A., Varley A., Gao G., Langer R., Xue W., Anderson D. (2023). Combinatorial design of nanoparticles for pulmonary mRNA delivery and genome editing. Nat. Biotechnol..

[69] Crudele J.M., Chamberlain J.S. (2018). Cas9 immunity creates challenges for CRISPR gene editing therapies. Nat. Commun..

