# Supplementary material for: A Versatile Reporter Platform for Evaluating HDR- and NHEJ-Based Genome Editing in Airway Epithelial Cell Cultures Using an rAAV Vector
Source: Viruses. 2025 Jun 6;17(6):821. doi: 10.3390/v17060821 (PMC12197413; doi:10.3390/v17060821)
Supplement: Supplementary file 1 [file viruses-17-00821-s001.zip › viruses-3667957-supplementary.pdf]

Supplementary Data

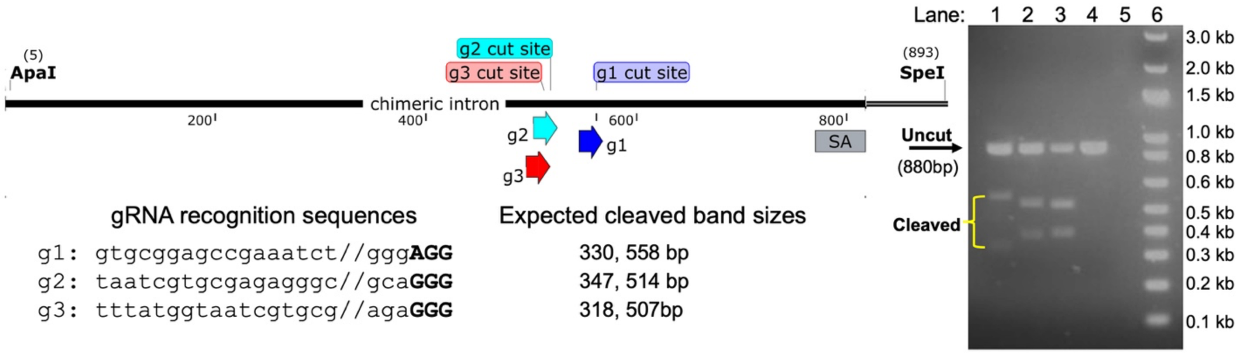

Supplementary Figure S1. Validation of candidate gRNAs by in vitro cleavage assay. gRNAs CAG-g1, CAG-g2 and CAG-g3 target distinct sequences (g1, g2 and g3, respectively) within the chimeric intron of the CAG promoter. An 880 bp DNA fragment (ApaI-SpeI, as depicted) was used as the substrate for *in vitro* cleavage assay by nucleoprotein (RNP) complexes comprising recombinant Cas9 protein and individual gRNAs. After incubation and proteinase K digestion, reactions were resolved on a 1% agarose gel and visualized via ethidium bromide (EtBr) staining. Among the three, RNP with gRNA CAG-g3 demonstrated the highest cleavage activity and was selected for HITI vector construction. Lane 1-3: Incubations of the DNA substrate with Cas9 complexed with CAG-g1 (Lane 1), GAG-g2 (Lane 2), and CAG-g3 (Lane 3); Lane 4, empty; Lane 5: Incubation of the DNA substrate and Cas9 protein only (no gRNA control); Lane 6: 1kb-plus DNA ladder (Invitrogen).

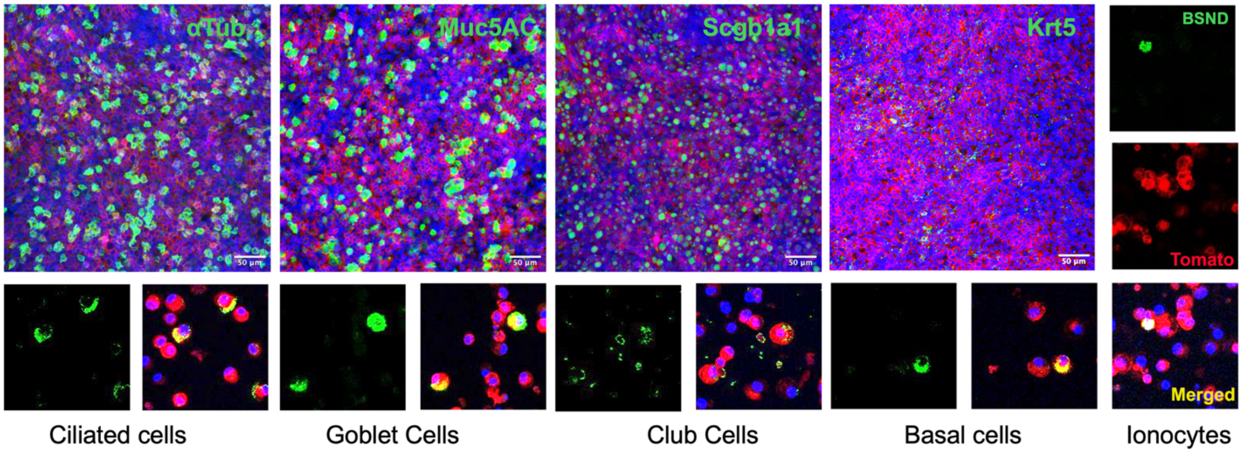

Supplementary Figure S2. Immunofluorescent analyses of epithelial cell types in polarized ferret airway epithelium cultured at an air-liquid interface (ALI). Airway basal cells were isolated from the trachea of a ROSA^{mTmG} ferret, enriched, and expanded in PneumaCult-Ex^{TM} plus medium. The cells were then cultured and differentiated at an ALI for three weeks using PneumaCult-ALI^{TM} medium. ALI cultures with transepithelial electrical resistance (TEER) > 1,000Ω/cm^2 were selected for immunofluorescent analyses. Immunostaining was performed directly on intact ALI cultures in the Transwell® and on cytospin slides prepared with dissociated cells from ALI cultures. Images were acquired using a Zeiss LSM 880 confocal microscope (objective: Plan-Apochromat 20 x /0.8 M27). Cell types and their corresponding marker antibodies are indicated. Due to their rarity, only images of cytospin preparation are shown for ionocytes. Primary antibodies used: aTub: anti-acetylated Tubulin (1:500; T7451, Sigma-Aldrich, St. Louis, MO), Muc5AC: anti-Mucin 5AC (1:500; MS-145-P, Thermo Fisher, Waltham, MA); SCGB1A1: anti-SCGB1A1 (anti-Uteroglobin, 1:2000; ABS1673, Millipore, St. Louis, MO); Krt5: anti-Keratin 5 (1:500; 905501, BioLegend, San Diego, CA), BSND (barttin CLCNK type accessory subunit beta): anti-BSND (1:500; ab196017, Abcam, Waltham, MA). DAPI was used to visualize nuclei (blue).

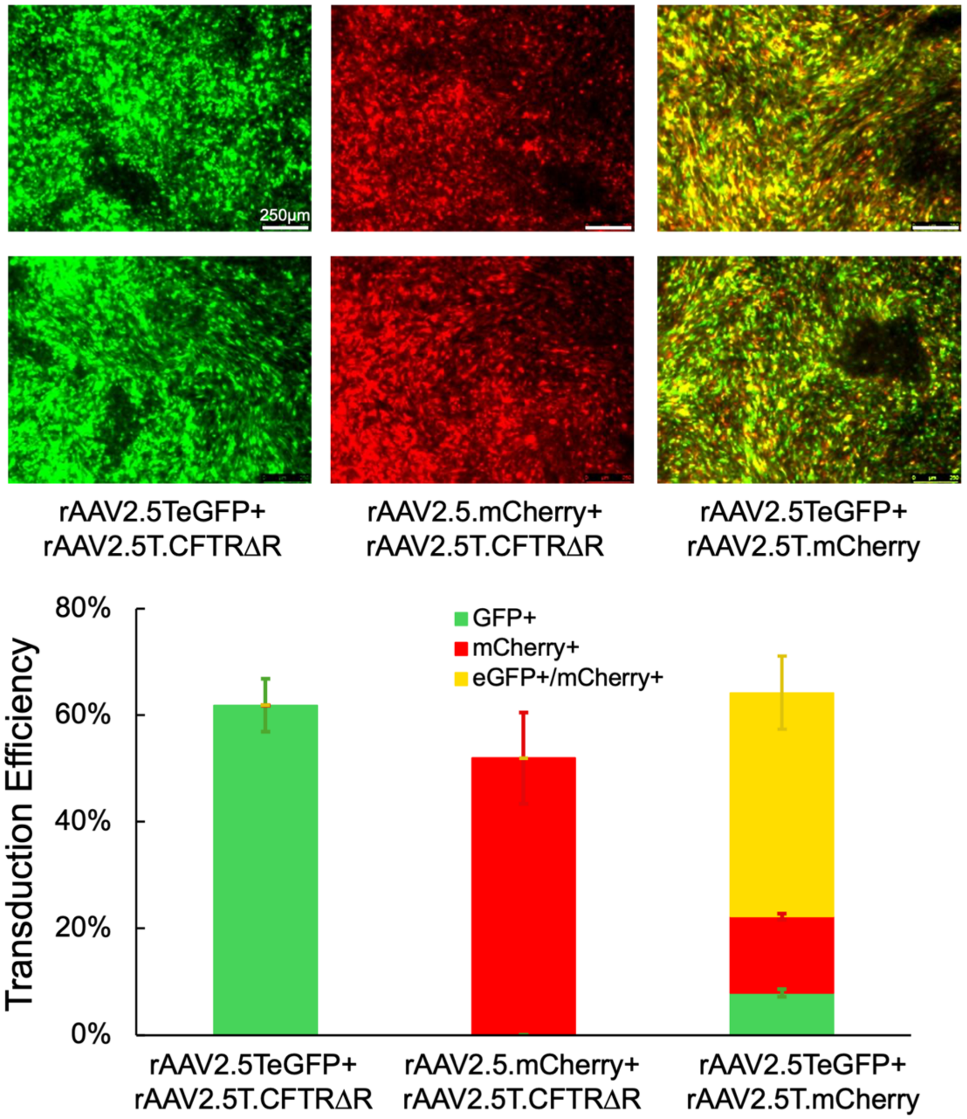

Supplementary Figure S 3. Dual rAAV2.5T co-transduction in well-differentiated CuFi-8 epithelial cultures. CuFi-8 ALI cultures were apically co-transduced with two indicated rAAV2.5T vectors at an MOI of 25K per vector, in the presence of 2.5 μM Doxorubicin. Representative fluorescence images were captured 9 days post-transduction prior to flow cytometry analysis. The percentage of eGFP-positive cells, mCherry-positive cells and the double-positive cells for both reporters were quantified to assess co-transduction efficiency. Data represent the mean ± standard deviation (SD) from n = 3 independent transductions. While only single-reporter expression (eGFP or mCherry) was observed in cultures co-transduced with a fluorescent reporter vector and a CFTR vector, co-transduction with both fluorescent reporter vectors resulted in 49.9 ± 6.36% eGFP-positive cells, 56.4 ± 6.66% mCherry-positive cells, and 42 ± 6.88% double-positive cells, indicating a high rate of co-delivery (79.1%) using the dual-AAV strategy.
